# Nasal Septal Deviations: A Systematic Review of Classification Systems

**DOI:** 10.1155/2016/7089123

**Published:** 2016-01-11

**Authors:** Jeffrey Teixeira, Victor Certal, Edward T. Chang, Macario Camacho

**Affiliations:** ^1^Department of Otolaryngology Head and Neck Surgery, Walter Reed National Military Medical Center, Bethesda, MD, USA; ^2^Department of Otorhinolaryngology/Sleep Medicine Centre, Hospital CUF, 4100-180 Porto, Portugal; ^3^Centre for Research in Health Technologies and Information Systems (CINTESIS), University of Porto, 4200-450 Porto, Portugal; ^4^Tripler Army Medical Center, Department of Surgery, Division of Otolaryngology, Tripler AMC, Honolulu, HI, USA; ^5^Tripler Army Medical Center, Division of Otolaryngology, Sleep Surgery and Sleep Medicine, 1 Jarrett White Road, Tripler AMC, HI 96859, USA

## Abstract

*Objective.* To systematically review the international literature for internal nasal septal deviation classification systems and summarize them for clinical and research purposes.* Data Sources.* Four databases (including PubMed/MEDLINE) were systematically searched through December 16, 2015.* Methods.* Systematic review, adhering to PRISMA.* Results.* After removal of duplicates, this study screened 952 articles for relevance. A final comprehensive review of 50 articles identified that 15 of these articles met the eligibility criteria. The classification systems defined in these articles included C-shaped, S-shaped, reverse C-shaped, and reverse S-shaped descriptions of the septal deviation in both the cephalocaudal and anteroposterior dimensions. Additional studies reported use of computed tomography and categorized deviation based on predefined locations. Three studies graded the severity of septal deviations based on the amount of deflection. The systems defined in the literature also included an evaluation of nasal septal spurs and perforations.* Conclusion.* This systematic review ascertained that the majority of the currently published classification systems for internal nasal septal deviations can be summarized by C-shaped or reverse C-shaped, as well as S-shaped or reverse S-shaped deviations in the anteroposterior and cephalocaudal dimensions. For imaging studies, predefined points have been defined along the septum. Common terminology can facilitate future research.

## 1. Introduction

Nasal septal deviations play a critical role in nasal obstruction symptoms, aesthetic appearance of the nose, increased nasal resistance, and sometimes snoring [1]. Consequently, a comprehensive assessment of the nasal septum serves an essential role in preoperative planning, reestablishing function, and overall cosmetic appeal. Typically, a septoplasty suffices in addressing significant nasal septal deviations, but on occasion such deviations warrant a single-stage septorhinoplasty [2–4]. In 1954, Lindahl described nasal septal deviations as either developmental (usually smooth, “C-shaped” or “S-shaped” nasal septum with occurrence more often in the anterior septum) or traumatic (usually irregular, angulated, and sometimes dislocated) in origin [5].

Over the years, individual authors and groups studied the assessment and classification of internal nasal septal deviations but none to date conducted a systematic evaluation of these studies with a comprehensive summarization of the individual results. Because of the variation in classification systems, such as grading internal septal deflections in the anterior aspect versus along the entire nasal airway, utilizing physical examination versus computed tomography, a summary of the currently published international literature would help facilitate future research. The importance of a summary is notable when studies report the prevalence of nasal septal deviations, given that studies reporting findings by simply using a handheld otoscope will have a lower prevalence of nasal septal deviations than those that use endoscopy or computed tomography because the handheld otoscope fails to consider the internal nasal septum's entire length and subsequently undercategorized these deviations. This in turn led to underestimations of the true prevalence of nasal septal deviations [6]. In order to facilitate future research regarding the effect of internal nasal septal deviations with regard to both functional (nasal obstruction) and cosmetic outcomes, a summary of currently available methods for categorizing nasal septal deviations is a necessary first step. The objective of this study is to conduct a systematic review of internal nasal septal deviation classification systems that are currently published in the international literature and summarize them for both clinical and research purposes.

## 2. Methods

The authors (Macario Camacho and Jeffrey Teixeira) conducted a systematic review of the literature found within PubMed/MEDLINE, Scopus, Web of Science, and the Cochrane Library from inception of the respective databases through December 16, 2015. Various searches identified the pertinent articles in the current published literature. The authors used the following MeSH terms: “Nose Deformities, Acquired” or “Nose” in combination with search terms “classification,” “classification system,” “grading,” and “grading system.” A second search used the MeSH terms “Nose Deformities, Acquired” and “Classification.” An additional search used the following phrases: “deviated nasal septum”; further searches used the following terms: “septal deviation” or “nasal deformity” in combination with search terms “classification,” “classification system,” “grading,” and “grading system.” The authors also reviewed “related citations” and “cited by” for relevant articles in order to identify additional possible studies to include. Further, the authors reviewed references cited within each article in order to identify additional studies.

The authors performed the literature search and independently reviewed the results to screen for relevant articles to include in the final review. The inclusion criteria included the following: studies which classify or grade internal nasal septal deviations, without regard to language. Exclusion criteria included the following: studies which do not classify or grade internal nasal septal deviations, or classification systems exclusively describing external nasal deformities. Statistical analysis was not applicable since there were no quantitative outcomes assessed, as this is a qualitative systematic review of classification systems. As part of the systematic review, the classification systems identified were each reviewed for commonalities, which could then be used to summarize the various classifications of nasal septal deviations.

## 3. Results

### 3.1. Study Selection

**Figure 1:**
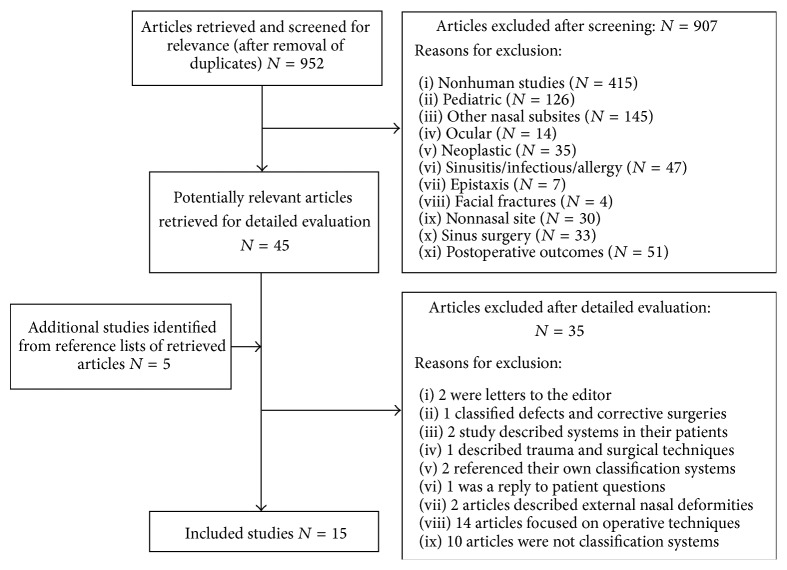
Flow diagram for the literature search and overall study selection.

An example of a search in PubMed is (((“Nose Deformities, Acquired” [Mesh]) OR “Nose” [Mesh]) AND “Classification” [Mesh]) OR (((“Nose Deformities, Acquired” [Mesh]) OR “Nose” [Mesh]) AND “Classification system”) OR (((“Nose Deformities, Acquired” [Mesh]) OR “Nose” [Mesh]) AND “Grading”) OR (((“Nose Deformities, Acquired” [Mesh]) OR “Nose” [Mesh]) AND “Grading System”) which yielded 541 results. Additional searches applied to all the databases produced a grand total of 952 articles, and after reviewing the titles and the abstracts a total of 907 articles were excluded. Full texts were downloaded for 45 articles [2–46]. After retrieval and reviewing the references of those articles, an additional five articles were downloaded [19, 47–50]. Of the 50 articles reviewed in their entirety, the following are reasons for exclusion: two were letters to the editor [13, 49], one classified nasal defects based on subunits and corrective surgeries [24], one study correlated previously described systems in their patients [37], one used a previously published classification system [46], one described trauma and surgical techniques [2], two referenced their own previously described classification system [21, 28], one was a questions and answers article [17], two articles focused on the external nasal deformities [15, 39], fourteen articles focused on operative techniques [4, 18–20, 29–32, 35, 36, 38, 40, 47, 50], and ten articles failed to meet criteria as classification systems [5, 7, 9–12, 14, 22, 34, 42]. A total of fifteen articles met inclusion criteria for describing internal nasal septal deviation classification systems [3, 6, 8, 16, 23, 25–27, 33, 41, 43, 44, 48, 51].  Figure 1 demonstrates the flow diagram for study selection.

### 3.2. Individual Study Results

Salihoglu et al. included 9,835 patients in their study evaluating the effect of nasal examination, including nasal septal deviations (graded as 1, 2, and 3 based on 33% increments), on nasal obstruction using the visual analog scale (VAS) [45]. In their study, they noted that nearly half of the patients had nasal septal deviations and of those about 60% were bilateral and 40% were unilateral [45]. Vidigal et al. used a nasal septal deviation classification based on the relationship of the nasal septum to the inferior turbinate [41]. Degree I: the deviation did not reach the inferior turbinate, degree II: the deviation reached the inferior turbinate, and degree III: the deviation reached the lateral wall and compressed the inferior turbinate [41]. Several authors reported classification systems that focus on common deviation patterns, including septal tilt, S-shaped deviation, and C-shaped deviations. Lawson in 1978 proposed a classification of the twisted nose into two basic types: the C shaped and S-shaped twist [51]. That study placed a focus on identifying skeletal asymmetries secondary to nasal bone fractures [51]. Guyuron et al. divided septal deviations into six classes to include C- and S-shaped deviations in the anteroposterior and cephalocaudal direction as well as localized deviation with nasal spur and septal tilt [16]. Cerkes classified nasal deviations into five categories to include caudal nasal septal deviations (classic septal tilt), anteroposterior C-shaped deviation, cephalocaudal C-shaped deviation, anteroposterior S-shaped deviation, and cephalocaudal S-shaped deviation [33]. Similar to Guyuron's and Cerke's classification system, Lee and Baker described S- and C-shaped deviations in the vertical and horizontal plane [43]. In this instance, Lee and Baker equated vertical direction to cephalocaudal direction and horizontal plane to anteroposterior [43]. Rohrich et al. classified internal nasal septal deviations based on three broad categories to include caudal septal deviations, concave dorsal deviations, and concave/convex dorsal deviations (S-shaped) [3]. The authors further divided caudal septal deviations and concave dorsal deformity into subtypes: straight septal tilt, concave deformity and S-shaped deformity for caudal septal deviation, and C-shaped dorsal deformity and reverse C-shaped dorsal deformity for concave dorsal deformity [3]. Parrilla et al. described corrective techniques for C- and S-shaped deformities and therefore by association recognized that these specific deformities exist, are reproducible, and require specific operative approach [4].

Rao et al. and Mladina used a similar classification system with a very precise description of the most common types of deviations seen in their practice [23, 27]. Mladina categorized the deviations into 7 types: Type 1: unilateral vertical septal ridge in the valve region that does not reach the valve itself, Type 2: unilateral vertical septal ridge in the valve region touching the nasal valve, Type 3: unilateral vertical ridge located more deeply in the nasal cavity, Type 4: S-shaped, Type 5: Almost horizontal septal spur, Type 6: massive unilateral bone spur, and Type 7: variation of these types [27]. Rao also classified septal deviations into 7 types: Type I: midline septum or mild deviations in vertical or horizontal plane, Type II: anterior vertical deviation, Type III: posterior vertical deviation, Type IV: S-septum, Type V: Horizontal spur on one side, Type VI: type V with a deep grove on the concave side, and Type VII: combination of II–VI [23].

**Figure 2:**
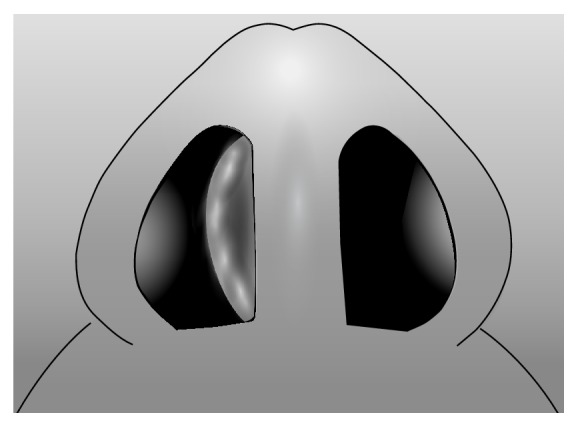
C-shaped nasal septal deviation in superoinferior dimension. Note: reverse C-shaped nasal septal deviation is the mirror image.

**Figure 3 fig3:**
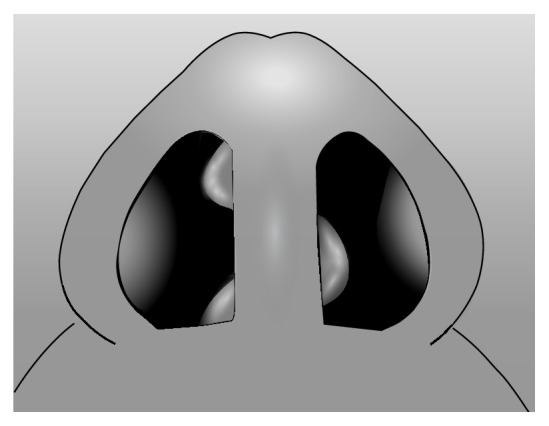
S-shaped nasal septal deviation in the superoinferior dimension. Note: reverse S-shaped nasal septal deviation is the mirror image.

**Figure 4 fig4:**
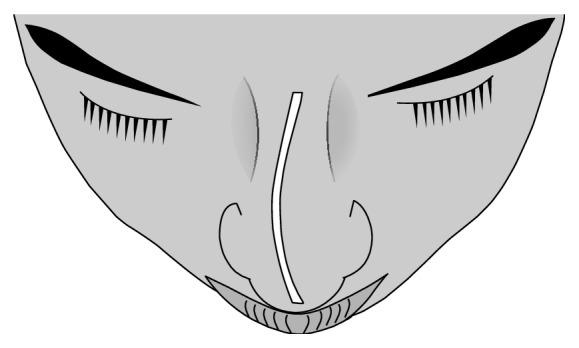
Internal and/or external nasal deviation in the anteroposterior dimension, C-shaped. Note: reverse C-shaped nasal septal deviation would be a mirror image.

**Figure 5 fig5:**
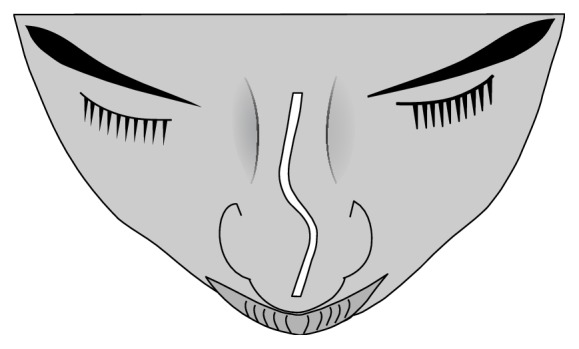
Internal and/or external nasal deviation in the anteroposterior dimension, S-shaped. Note: reverse S-shaped nasal septal deviation would be a mirror image.

**Figure 6 fig6:**
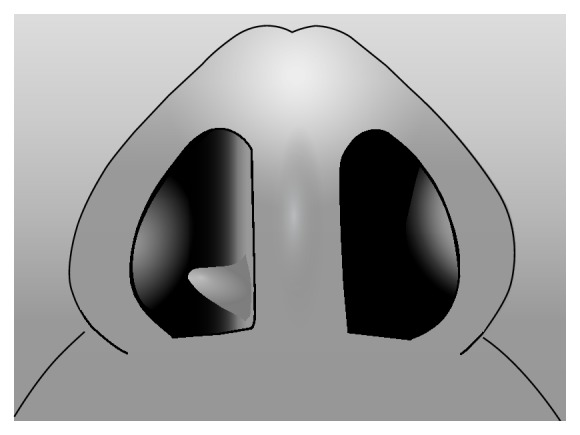
Outpouching (nasal septal spur).

I. Baumann and H. Baumann classified types of septal deviation into 6 types, where each type has several additional features: Type 1: septal crest, Type 2: cartilaginous deviated nose, Type 3: high septal crest deviation, Type 4: caudally inclined septum, Type 5: septal crest, and Type 6: caudally inclined septum [25]. Jin et al. followed a very similar format to Rao and Mladina by proposing four types of septal deviations: Type I: localized deviation including spur (spine), crest, or caudal dislocation, Type II: curved/angulated deviation without localized deviation, Type III: curved/angulated deviation with localized deviation, and Type IV: curved/angulated deviation with associated external nasal deformity [26]. However, the authors further described septal deviation by including anatomic site as well as severity of septal deviation (mild, moderate, and severe) [26]. Sawhney and Sinha also emphasized the importance of classifying nasal septal deviation by the severity of deviation (marked, moderate, and mild) [8]. They integrated the following within the level of severity: cartilage and bony deflection, dislocation of septal cartilage, and level of deviation [8]. Buyukertan et al. divided the internal nasal septum into 10 segments: anterosuperior (AS), anteromedial (AM), anteroinferior (AI), mediosuperior (MS), mediomedia (MM), medioinferior (MI), posterosuperior (PS), posteromedial (PM), posteroinferior (PI), and caudal end (CE) of the septum nasi [48]. Lin et al. selected points on computed tomography imaging and analyzed them by computer software that compared contours of a deviated septum as compared to an ideal straight septum [44]. Points assessed included the perpendicular plate of the ethmoid bone and vomer bone junction, nasal spine, nasal bone, crista galli, and midpoint between the perpendicular plate-vomer junction and nasal spine [44].  Table 1 summarizes the various internal nasal septal deviation classification systems identified in this review, and Figures 2–7 demonstrate the combined internal classification systems.

## 4. Discussion

Septal deviations play a crucial role in functional nasal breathing. Unrecognized internal nasal septal deviations stand as the primary reason for failed rhinoplasty outcomes due to the pivotal role of the internal nasal septal deviation in migration and further deviation of nasal bones and lateral cartilage. Consequently, as many as 50% of cases of posttraumatic nasal deformity require subsequent revision rhinoplasty or septorhinoplasty [2, 4]. Parrilla et al. highlighted the importance in considering the anatomy behind the deviation and how preoperative nasal septal analysis guides the preoperative assessment and plan as well as operative technique, reducing the risk of complication and repeat surgery which in themselves present with cumbersome challenges [4].

As stressed in the literature, identification of the C- and S-shaped deformities at the time of planning remains crucial to identifying potentially complex surgeries compared to less technically challenging operative interventions such as septal tilts [25, 43]. C- and S-shaped deformities are sometimes surgically scored on the convex side to silence the cartilaginous memory and frequently enhanced with grafting material to support and straighten the cartilaginous septum [3, 43]. Perforations also present with unique challenges, as the repair of a septal perforation usually necessitates bilateral elevation of the surrounding mucoperichondrium. While a unilateral perforation typically heals with no surgical intervention, bilateral opposing perforations can lead to permanent septal perforation. Accurately identifying septal perforations further allows for appropriate preoperative planning including the need for grafting material versus flap.

Further, external nasal deformities and internal nasal septal deviations exist symbiotically. Accurately assessing these aspects and characteristics during the physical exam remains imperative to optimizing the assessment and preoperative planning process. Elicitation of a history of specific trauma and correlating the nuances of the injury with the specific findings on external exam and internal exam ensure accuracy of assessment. As pointed out by Cerkes, findings of anteroposterior C-shaped deviation correlate with an external deviation on the opposite side of the internal deviation while a cephalocaudal C-shaped deviation usually presents as a visible C-shaped external deformity [33]. Findings not consistent with such patterns should alert the clinician to other underlying forces that may be contributing to the noted deviation. Early identification of such forces and components allows for appropriate presurgical planning and therefore avoidance of surgical failure and further operative procedures. Evaluation of the external nose typically involves division of the nose into thirds with deviation of the upper third resulting from fractured nasal bones. Accurately identifying upper third deformities remains important, as correction of the septum alone typically fails to change the cosmetic appearance of the nose. Such deformities likely require osteotomies of the nasal bones for reapproximation and successful surgical management to improve the cosmetic appearance (i.e., rhinoplasty). However, the septum influences the middle and lower thirds of the nose to a greater degree with more emphasis on the latter. When imagining the biomechanics of the nose and septum, the quadrangular cartilage acts like a ridge board on a roof with the lateral cartilages functioning as the rafters. Consequently, an unstable quadrangular cartilage leads to unstable lateral cartilages contributing to external deformity or collapse. This analogy emphasizes the importance of assessing external deformity when evaluating the internal nasal septum.

Additionally, rhinomanometry and acoustic rhinometry evaluations reveal enhanced nasal resistance and diminished nasal volumes in patients with obstructive sleep apnea (OSA). Although a systematic review demonstrated that nasal findings have not been included in any of the 28 studies that used mathematical equations [52] to predict positive airway device treatment pressures (PAP pressures), it has been demonstrated in a meta-analysis that after nasal surgery (to include septoplasty) there is an associated decrease in PAP pressures and increased device use after septoplasty and/or additional nasal surgeries [53]. Vidigal et al. demonstrated that patients with OSA exhibit higher scores of nasal symptoms with higher frequency of complaints of nasal obstruction, nasal alterations, and inferior turbinate hypertrophy [41]. While acoustic rhinometric results appear statistically insignificant, the authors noted that rhinometric measurements fail to account for the dynamic changes of resistance, such as nasal cycling during wake and sleep [54]. Furthermore, acoustic rhinometry measurements demonstrated the greatest reproducible results in the first five centimeters (cm) of the nasal cavity, and as a result anterior deviations produce the greatest results while posterior deviations contribute little if anything to these measurements. To their credit, the authors of the study mentioned this discrepancy of failing to differentiate the deviations into posterior and anterior which likely affected their results [41]. As pointed out by Reitzen et al., turbinate hypertrophy as well as mucosal edema also appears to contribute to airflow resistance [6]. Preoperative identification of these contributing factors allows for categorization of additional areas of anatomical obstruction (inferior turbinate grades [55], nasal polyposis, etc.) and may demonstrate the need for additional procedures such as bilateral inferior turbinoplasty, sinus surgery, or a rhinoplasty.

## 5. Conclusion

This systematic review ascertained that the majority of the currently published classification systems for internal nasal septal deviations can be summarized by C-shaped or reverse C-shaped, as well as S-shaped or reverse S-shaped, deviations in the anteroposterior and cephalocaudal dimensions. For imaging studies predefined points have been defined along the septum. Common terminology can facilitate future research.

## Figures and Tables

**Figure 7:**
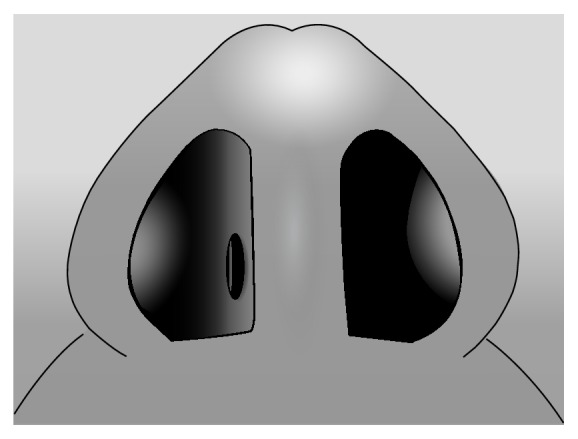
Open communication (nasal septal perforation).

**Table 1:** Studies meeting criteria for internal nasal septal deviation classification systems and a summary of the descriptions.

Author, year, location	Description of internal nasal septal deviation classification systems
Lin et al., 2014, USA [44]	Various points on computed tomography imaging which were analyzed by computer software that compared contours of a deviated septum as compared to an ideal straight septum. Points assessed included the perpendicular plate of the ethmoid bone and vomer bone junction, nasal spine, nasal bone, crista galli, and midpoint between the perpendicular plate-vomer junction and nasal spine.

Salihoglu et al., 2014, Turkey [45]	Grade 1: 0–33% deflection from midline toward lateral wall, Grade 2: 34–66% deflection from midline toward the lateral wall, and Grade 3: 67–100% deflection from the midline toward the lateral wall.

Vidigal et al., 2013, Italy [41]	Degree I: the deviation did not reach the lower nasal turbinate, Degree II: the deviation reached the lower nasal turbinate, and Degree III: the deviation reached the lateral wall and compressed the lower nasal turbinate.

Lee and Baker, 2013, USA [43]	Caudal septum is straight but deviated from the midline and is usually displaced from the maxillary crest, C-shaped septal deformity in the vertical plane, C-shaped septal deformity in the horizontal plane, S-shaped septal deformity in the horizontal plane, and S-shaped septal deformity in the vertical plane.

Reitzen et al., 2011, USA [6]	Tortuosity is measured at 4 defined points along the length of internal nasal septum. A ratio of the actual length of the septum (*T*) and the ideal length (*I*) is calculated as *T*/*I*.

Cerkes, 2011, Turkey [33]	Caudal septal deviation (septal tilt), anteroposterior C- and S-shaped deviation, and cephalocaudal C- and S-shaped deviations.

Jin et al., 2007, Korea [26]	Type I: localized deviation including spur (spine), crest, or caudal dislocation, Type II: curved/angulated deviation without localized deviation, Type III: curved/angulated deviation with localized deviation, and Type IV: curved/angulated deviation with associated external nasal deformity.

I. Baumann and H. Baumann, 2007, Germany [25]	Types based on primary deviation, each type has several additional features: Type 1: septal crest, Type 2: cartilaginous deviated nose, Type 3: high septal crest deviation, Type 4: caudally inclined septum, Type 5: septal crest, and Type 6: caudally inclined septum.

Rao et al., 2005, India [23]	Type I: midline septum or mild deviations in vertical or horizontal plane, Type II: anterior vertical deviation, Type III: posterior vertical deviation, Type IV: S-septum, Type V: horizontal spur on one side, Type VI: type V with a deep grove on the concave side, and Type VII: combination of II–VI.

Buyukertan et al., 2003, Turkey [48]	The septum is divided into 10 segments: anterosuperior (AS), anteromedial (AM), anteroinferior (AI), mediosuperior (MS), mediomedia (MM), medioinferior (MI), posterosuperior (PS), posteromedial (PM), posteroinferior (PI), and caudal end of the septum nasi (CE).

Rohrich et al., 2002, USA [3]	Caudal septal deviation (straight septal tilt, C-shaped, and S-shaped), concave dorsal deformity (C-shaped dorsal deformity and reverse C-shaped dorsal deformity), and concave/convex dorsal deformity (S-shaped).

Guyuron et al., 1999, USA [16]	C-shape anteroposterior deviation, C-shape cephalocaudal, S-shape anteroposterior, S-shape cephalocaudal, septal tilt deformity, and localized deviations or large spurs.

Mladina, 1987, Croatia [27]	Type 1: unilateral vertical septal ridge in the valve region that does not reach the valve itself, Type 2: unilateral vertical septal ridge in the valve region touching the nasal valve, Type 3: unilateral vertical ridge located more deeply in the nasal cavity, Type 4: S-shaped, Type 5: almost horizontal septal spur, Type 6: massive unilateral bone spur, and Type 7: variation.

Lawson, 1978, Canada [51]	C-shaped, S-shaped, and deviated nose, twisted nose, and skeletal asymmetry (depressed nasal fracture).

Sawhney and Sinha, 1964, India [8]	Grade deviations as mild, moderate, and marked (cannot see middle turbinate on side of the deviation). Cartilage and bony deflection, dislocation of septal cartilage, and level of deviation.

## References

[1] Hsia J. C., Camacho M., Capasso R. (2014). Snoring exclusively during nasal breathing: a newly described respiratory pattern during sleep. *Sleep & Breathing*.

[2] Higuera S., Lee E. I., Cole P., Hollier L. H., Stal S. (2007). Nasal trauma and the deviated nose. *Plastic and Reconstructive Surgery*.

[3] Rohrich R. J., Gunter J. P., Deuber M. A., Adams W. P. (2002). The deviated nose: optimizing results using a simplified classification and algorithmic approach. *Plastic and Reconstructive Surgery*.

[4] Parrilla C., Artuso A., Gallus R., Galli J., Paludetti G. (2013). The role of septal surgery in cosmetic rhinoplasty. *Acta Otorhinolaryngologica Italica*.

[5] Lindahl J. W. (1954). The deviated nasal septum. *The Practitioner*.

[6] Reitzen S. D., Chung W., Shah A. R. (2011). Nasal septal deviation in the pediatric and adult populations. *Ear, Nose and Throat Journal*.

[7] Gibb A. G. (1963). Deviated nasal septum. *Nursing Times*.

[8] Sawhney K. L., Sinha A. (1964). Diagnosis of deviated nasal septum. *Journal of the Oto-laryngological Society of Australia*.

[9] Birrell J. F. (1970). Nasal polypi and the deviated nasal septum. *The Practitioner*.

[10] Iliades C. E. (1973). The deviated nasal septum indications for sub mucous resection. *Eye, Ear, Nose & Throat Monthly*.

[11] Conrad K. (1978). Correction of crooked noses by external rhinoplasty. *The Journal of Otolaryngology*.

[12] Jaffe B. F. (1981). Classification and management of anomalies of the nose. *Otolaryngologic Clinics of North America*.

[13] Bookman R. (1983). Deviated nasal septum as a diagnosis. *Annals of Allergy*.

[14] Matschke R. G., Fiebach A. (1985). Septum deviation and concomitant sinusitis. *HNO*.

[15] Ellis D. A. F., Gilbert R. W. (1991). Analysis and correction of the crooked nose. *The Journal of Otolaryngology*.

[16] Guyuron B., Uzzo C. D., Scull H. (1999). A practical classification of septonasal deviation and an effective guide to septal surgery. *Plastic and Reconstructive Surgery*.

[17] (2000). What's a deviated nasal septum? Does it need to be corrected?. *Mayo Clinic Health Letter*.

[18] Becker D. G. (2003). Septoplasty and turbinate surgery. *Aesthetic Surgery Journal*.

[19] Gunter J. P., Rohrich R. J. (1988). Management of the deviated nose. The importance of septal reconstruction. *Clinics in Plastic Surgery*.

[20] Byrd H. S., Salomon J., Flood J. (1998). Correction of the crooked nose. *Plastic and Reconstructive Surgery*.

[21] Guyuron B., Behmand R. A. (2003). Caudal nasal deviation. *Plastic and Reconstructive Surgery*.

[22] Egeli E., Demirci L., Yazýcý B., Harputluoglu U. (2004). Evaluation of the inferior turbinate in patients with deviated nasal septum by using computed tomography. *Laryngoscope*.

[23] Rao J. J., Kumar E. C. V., Babu K. R., Chowdary V. S., Singh J., Rangamani S. V. (2005). Classification of nasal septal deviations—relation to sinonasal pathology. *Indian Journal of Otolaryngology and Head and Neck Surgery*.

[24] Bayramiçli M. (2006). A new classification system and an algorithm for the reconstruction of nasal defects. *Journal of Plastic, Reconstructive and Aesthetic Surgery*.

[25] Baumann I., Baumann H. (2007). A new classification of septal deviations. *Rhinology*.

[26] Jin H. R., Lee J. Y., Jung W. J. (2007). New description method and classification system for septal deviation. *Journal of Rhinology*.

[27] Mladina R. (1987). The role of maxillar morphology in the development of pathological septal deformities. *Rhinology*.

[28] Mladina R., Čujić E., Šubarić M., Vuković K. (2008). Nasal septal deformities in ear, nose, and throat patients: an international study. *American Journal of Otolaryngology: Head and Neck Medicine and Surgery*.

[29] Seyhan A., Ozaslan U., Sir E., Ozden S. (2008). Three-dimensional modeling of nasal septal deviation. *Annals of Plastic Surgery*.

[30] Dobratz E. J., Park S. S. (2009). Septoplasty pearls. *Otolaryngologic Clinics of North America*.

[31] Haack J., Papel I. D. (2009). Caudal septal deviation. *Otolaryngologic Clinics of North America*.

[32] Foda H. M. T. (2010). The crooked nose: correction of dorsal and caudal septal deviations. *HNO*.

[33] Cerkes N. (2011). The crooked nose: principles of treatment. *Aesthetic Surgery Journal*.

[34] Godoy A., Ishii M., Byrne P. J., Boahene K. D. O., Encarnacion C. O., Ishii L. E. (2011). The straight truth: measuring observer attention to the crooked nose. *Laryngoscope*.

[35] Shipchandler T. Z., Papel I. D. (2011). The crooked nose. *Facial Plastic Surgery*.

[36] Sykes J. M., Kim J.-E., Shaye D., Boccieri A. (2011). The importance of the nasal septum in the deviated nose. *Facial Plastic Surgery*.

[37] Sam A., Deshmukh P. T., Patil C., Jain S., Patil R. (2012). Nasal septal deviation and external nasal deformity: a correlative study of 100 cases. *Indian Journal of Otolaryngology and Head and Neck Surgery*.

[38] Boccieri A. (2013). The crooked nose. *Acta Otorhinolaryngologica Italica*.

[39] Jang Y. J., Wang J. H., Lee B.-J. (2008). Classification of the deviated nose and its treatment. *Archives of Otolaryngology—Head & Neck Surgery*.

[40] Cho G. S., Jang Y. J. (2013). Deviated nose correction: different outcomes according to the deviation type. *The Laryngoscope*.

[41] Vidigal T. D. A., Haddad F. L. M., Gregório L. C., Poyares D., Tufik S., Bittencourt L. R. A. (2013). Subjective, anatomical, and functional nasal evaluation of patients with obstructive sleep apnea syndrome. *Sleep & Breathing*.

[42] Karataş D., Yüksel F., Şentürk M., Dogan M. (2013). The contribution of computed tomography to nasal septoplasty. *The Journal of Craniofacial Surgery*.

[43] Lee J. W., Baker S. R. (2013). Correction of caudal septal deviation and deformity using nasal septal bone grafts. *JAMA Facial Plastic Surgery*.

[44] Lin J. K., Wheatley F. C., Handwerker J., Harris N. J., Wong B. J. F. (2014). Analyzing nasal septal deviations to develop a new classification system: a computed tomography study using MATLAB and OsiriX. *JAMA Facial Plastic Surgery*.

[45] Salihoglu M., Cekin E., Altundag A., Cesmeci E. (2014). Examination versus subjective nasal obstruction in the evaluation of the nasal septal deviation. *Rhinology*.

[46] Prasad S., Varshney S., Bist S. S., Mishra S., Kabdwal N. (2013). Correlation study between nasal septal deviation and rhinosinusitis. *Indian Journal of Otolaryngology and Head and Neck Surgery*.

[47] Francesconi G., Fenili O. (1973). Treatment of deflection of the anterocaudal portion of the nasal septum. *Plastic and Reconstructive Surgery*.

[48] Buyukertan M., Keklikoglu N., Kokten G. (2003). A morphometric consideration of nasal septal deviations by people with paranasal complaints; a computed tomography study. *Rhinology*.

[49] Mladina R. (2003). A morphometric consideration of nasal septal deviations by people with paranasal complaints; A computed tomography study. *Rhinology*.

[50] Cottle M. H., Loring R. M., Fischer G. G., Gaynon I. E. (1958). The maxilla-premaxilla approach to extensive nasal septum surgery. *A.M.A. Archives of Otolaryngology*.

[51] Lawson V. G. (1978). Management of the twisted nose. *The Journal of Otolaryngology*.

[52] Camacho M., Riaz M., Tahoori A., Certal V., Kushida C. A. (2015). Mathematical equations to predict positive airway pressures for obstructive sleep apnea: a systematic review. *Sleep Disorders*.

[53] Camacho M., Riaz M., Capasso R. (2015). The effect of nasal surgery on continuous positive airway pressure device use and therapeutic treatment pressures: a systematic review and meta-analysis. *Sleep*.

[54] Kimura A., Chiba S., Capasso R. (2013). Phase of nasal cycle during sleep tends to be associated with sleep stage. *The Laryngoscope*.

[55] Camacho M., Zaghi S., Certal V. (2015). Inferior turbinate classification system, grades 1 to 4: development and validation study. *The Laryngoscope*.

